# Reconstitution of immune cell in liver and lymph node of adult- and newborn-engrafted humanized mice

**DOI:** 10.1186/s12865-016-0157-9

**Published:** 2016-06-16

**Authors:** Crystal Dykstra, Amanda J. Lee, Evan J. Lusty, Mira M. Shenouda, Mahsa Shafai, Fatemeh Vahedi, Marianne V. Chew, Stephen Collins, Ali A. Ashkar

**Affiliations:** Department of Pathology and Molecular Medicine, McMaster Immunology Research Centre, Hamilton, ON L8N 3Z5 Canada; Department of Medicine, McMaster University, Hamilton, ON L8N 3Z5 Canada; McMaster University, 1280 Main Street West, MDCL 4015, Hamilton, ON Canada

**Keywords:** Humanized mice, Liver, Natural killer cells

## Abstract

**Background:**

Humanized mouse models are an increasingly popular preclinical model to study the human immune response in a biological system. There are a variety of protocols to generate these mice, each differing in the strain of the recipient, source of hematopoietic stem cells, and mode of transplantation. Though there is well-documented reconstitution information regarding the spleen, blood, and bone marrow, there is little information regarding reconstitution of the lymph node and liver. In this report, we sought to compare reconstitution levels in a variety of immunological tissues, including the lymph node and liver, between mice engrafted intravenously as adults and intrahepatically in newborns.

**Results:**

CD34+ cells were enriched from cord blood and transplanted intravenously into irradiated adult NOD-Rag1^-/-^IL2rγ^-/-^ (NRG) mice or intra-hepatically into irradiated newborn NRG mice. At 9–28 weeks post-engraftment, immunological tissues were processed and analyzed for human lymphoid and myeloid subsets. Adult and newborn engrafted humanized mice were comparable in long-term reconstitution of human CD45 cells and subsequent lymphoid and myeloid subsets in the spleen, bone marrow, thymus, lymph node, and liver. Mice engrafted as newborns had a higher level of T-cells and a lower level of B-cells compared to mice engrafted as adults. We observed significant levels of human immune cell engraftment in both the lymph node and the liver, with a predominant adaptive immune population in both compartments.

**Conclusions:**

Human immune cells repopulate liver and mesenteric lymph nodes of NRG mice and can be used to study the human immune system in the gastrointestinal tract.

**Electronic supplementary material:**

The online version of this article (doi:10.1186/s12865-016-0157-9) contains supplementary material, which is available to authorized users.

## Key points

- Mice humanized as newborns or adults display comparable levels of human immune cell reconstitution
- We observed significant levels of human immune cell reconstitution in the mesenteric lymph node and liver
- Each immunological organ has a distinct human immune profile in the humanized mouse model
- Human NK cells in the livers of humanized mice display a decreased activation and maturation phenotype compared to peripheral human NK cells

## Background

Although many human pathogens currently under research have been adapted to the mouse model, some are strictly human pathogens [1]. Further, mice are evolutionarily distinct from humans and are short-lived [1]. Within the last 30 years, however, researchers have been developing and improving upon a hybrid mouse model wherein human tissues are housed in the background of a mouse. The generation of the humanized mouse model now allows researchers to directly study the dynamics of human immune cells in a biological system.

The first humanized mouse models engrafted a human immune system into SCID mice [1, 2]. Since then, many protocols have used immunocompromised mice with varying knockout combinations of NOD, SCID, IL-2R, Rag1, and Rag2 genes, wherein the complete absence of all lymphocytes has thus far yielded the best human immune cell engraftment efficiency [1, 2]. Furthermore, there are a variety of hematopoietic stem cells sources (HSCs; i.e. peripheral blood, fetal liver, and cord blood) along with a number of different methods to engraft those progenitor cells, such as intrahepatically or intraperitoneally into newborn mice or intravenously into adult mice. Each method tends to yield different reconstitution rates and immune cell distributions [1, 2]. In regards to HSCs, progenitor CD34+ cells from cord either blood or fetal liver are a superior HSC source compared to PBMCs or bone marrow as there is a higher level of engraftment and limited development of graft vs. host disease [3].

A large variety of protocols for generating humanized mice now exist and encompass an array of murine recipients with different sources of HSCs and the methods of engraftment. This results in varying reports of human immune cell engraftment efficiencies and human immune cell subset distribution [1, 2]. In particular, there is very little data comparing the engraftment of HSCs into mice as newborns or as adults. Interestingly, Brehm et al. [4] has shown that mice engrafted as newborn mice have an overall greater reconstitution of human CD45+ cells in the blood compared to those engrafted as adults. Furthermore, newborn engrafted mice had a greater proportion of T-cells and a lower proportion of B-cells in the blood compared to mice engrafted as adults [4]. Unfortunately, the comparison between mice engrafted as newborns or adults was limited to the blood and it is unknown whether this trend prevails in all other immunological organs, including the liver.

Much of the data analyzing human immune cell reconstitution in mice has been limited to the blood, spleen, bone marrow, and thymus. Little is know about human immune cells in lymph nodes or liver. Ishikawa et al. [5] as well as Traggai et al. [6] both observed mesenteric lymph node development in humanized mice engrafted as newborns, though using different strains of recipient mice. Traggai et al. [6] further showed that the mesenteric lymph nodes contained both B and T-cells. Recently, researchers have developed a new humanized mouse model in which human liver cells are engrafted into immunocompromised mice, allowing for the study of human liver tropic viruses such as hepatitis C virus and malaria, with the ultimate goal of creating a humanized mouse model with both human liver and a human immune system [7–9]. However, the human immune cell composition in the livers of humanized mice is not well characterized.

In this article, we have conducted a comprehensive comparative analysis of human immune cell engraftment and distribution between mice given HSCs as newborn pubes (1–2 days old) intrahepatically or intravenously as adult mice in a variety of organs, including the lymph node and liver.

## Methods

### Ethics

All animal experiments were approved by the Animal Research Ethic Board of McMaster University. Use of human cord blood was approved by the Research Ethic Board of McMaster University. Human cord blood samples were obtained with written informed consent from the mother.

### Mice

NRG mice were purchased from the Jackson Lab (Bar Harbor, ME). The mice were bred and housed under specific pathogen-free conditions in a level II facility at the Central Animal Facility at McMaster University. Mouse colonies were maintained on a 12 h light/12 h dark cycle.

### HSC engraftment into NRG mice

Human umbilical cord blood was collected after delivery (Department of Obstetrics and Gynecology, McMaster Children’s Hospital, Hamilton, ON, Canada). Newborn pups and adults were engrafted as described in [10]. Human CD34+ HSCs were enriched by negative immunodepletion of CD2, CD3, CD14, CD16, CD19, CD24, CD56, CD66b and glycophorin A cells using a commercially available kit (RosetteSep, StemCell Technologies, Vancouver, BC, Canada), as per the manufacturer’s instructions. This was followed by Lymphoprep density gradient centrifugation. The isolated cells were cryopreserved in freezing media containing 90 % FBS and 10 % DMSO and then stored in liquid nitrogen. Newborn pups were irradiated using a gamma irradiator (Gammacell 3000) within 72 h of birth with two doses of 9 cGy separated by 3 h. Immediately after the second dose of irradiation, 1 × 10^6^ to 2 × 10^6^ thawed HSC cells or freshly isolated cells were resuspended in 30–40 μl of Phosphate Buffered Saline (PBS) and injected intrahepatically with a 30 g needle. The pups were then weaned 21 days of age. Adult NRG mice (6–10 weeks old) were irradiated with a single dose of 550 cGy. Similar to the pups, approximately 1 × 10^6^ to 2 × 10^6^ cells were injected immediately following irradiation of the mice via the lateral tail vein in a total volume of 300 μl diluted in PBS by a 30 g needle. All engrafted mice were facially bled 12 weeks following HSC engraftment. Twelve weeks following HSC engraftment, blood was analyzed for the human leukocyte reconstitution by assessment of human CD45+ cells. Mice with human CD45 levels greater than 15 % were selected for experiments.

#### Isolation of blood, bone marrow, thymus, spleen, liver and lymph node

Blood samples were taken from the facial vein before sacrificing the mice and isolation of all other tissues. The mice were euthanized and single cell suspensions of the samples were prepared by mechanical crushing, which was subsequently passed through a 40 μm nylon mesh cell strainer (BD) to remove aggregates and debris [10]. The liver cell suspension was subjected to a LymphoprepTM density gradient for isolation of the leukocyte population. Red blood cells were removed using Ammonium-Chloride-Potassium Lysing Buffer (Life Technologies, Grand Island, NY, USA).

### Flow cytometry analysis

Cell suspensions were resuspended in a 0.2 % bovine serum albumin PBS solution and were blocked with anti mouse CD16/CD132 antibody (eBiosciences, San Diego, CA). Samples were stained with the antibody panels described in Additional file 1: Table S1. Stained cells were analyzed on a LSR II Flow Cytometry System (BD Biosciences) and data was analyzed using FlowJo (Tree Star).

### Statistical analysis

Data are presented as average ± SD. Differences between groups were analyzed by one-way ANOVA and post-hoc Turkey’s Multiple Comparison Test. A *P* value <0.05 was considered statistically significant. All calculations were performed using the GraphPad Prism software package (Graphpad Software Inc., San Diego, CA).

## Results

### Intravenous injection in NRG adults and intrahepatic injection in NRG newborns results in similar levels of human CD45+ cell reconstitution

**Fig. 1:**
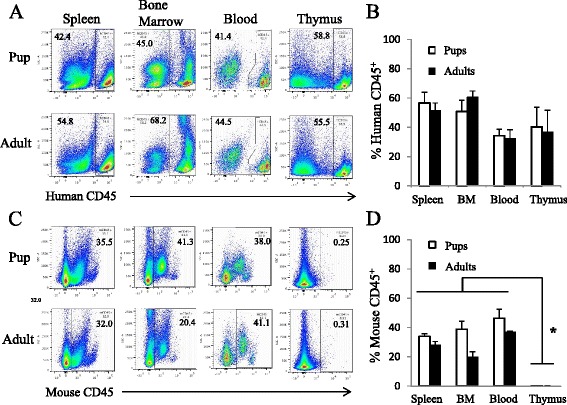
Similar levels of human immune cell reconstitution between NRG mice engrafted intravenously as adults or intrahepatically as pups with human CD34+ cells. NRG mice were engrafted with human CD34+ cells either intravenously as adults or intrahepatically as newborn pups. At 22 to 28 weeks after transplantation, spleen, bone marrow, blood and thymuses were taken from the engrafted NRG mice and examined for human and mouse CD45 expression. Representative flow plots of human and mouse CD45 expression in isolated tissues shown in (**a**) and (**c**), respectively. The percentage of human CD45+ cells in NRG engrafted mice are graphically represented in (**b**). Percentage of mouse CD45+ cells in NRG engrafted mice are graphically represented in (**d**). *n* = 3; *p < 0.05

We first compared reconstitution of human CD45+ cells between two different methods of humanized mice generation: intrahepatic injection into newborn pups or intravenous injection into adult NRG mice. At 12–28 weeks post engraftment, we observed a similar level of human immune cell reconstitution in the isolated tissues between the two methods, with higher levels of reconstitution found in the spleen and bone marrow (Fig. 1a and b). We also examined and compared the proportion of mouse CD45+ cells in the spleen, blood, bone marrow, and thymus between mice engrafted as adults and newborn pups. As expected, both groups of humanized mice had limited expression of mouse CD45+ cells in the thymus (Fig. 1c and d).

### Engraftment of adult NRG mice intravenously showed a higher proportion of CD19+ B-cells and lower proportion of CD3+ T-cells in the blood compared to engraftment of newborns intrahepatically

**Fig. 2:**
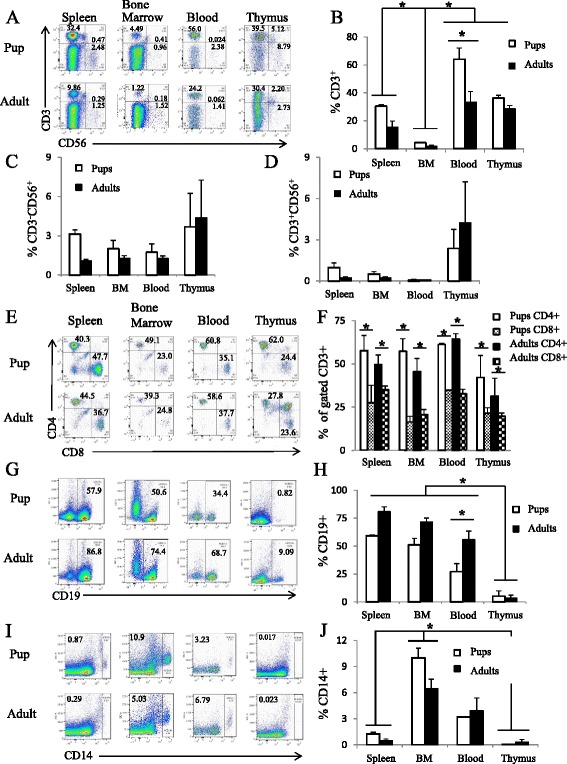
Differences in profile of human lymphoid and myeloid cell reconstitution between spleen, bone marrow, blood, and thymus. At 22 to 28 post-engraftment, spleen, bone marrow, blood, and thymus were isolated, processed, and examined for human CD45, CD3, CD4, CD8, CD56, CD14, and CD19 expression. All events were first gated on human CD45 expression and subsequently examined for T- and B-cell, NK cell, NKT cell, and myeloid cell-specific markers. Human CD45+ cells were first examined for CD3 and CD56 expression. Representative flow plots for each tissue are displayed in (**a**). Proportions of human CD3+ T-cells stratified by tissue shown in (**b**). Proportions of human CD3-CD56+ NK cells shown in (**c**). Proportions of human CD3 + CD56+ NKT cells graphically shown in (**d**). Human CD3+ cells were then examined for human CD4+ and CD8+ expression. Representative flow plots shown in (**e**) and proportions of human CD4+ and CD8+ T-cells within each tissue are shown in (**f**). Human CD45+ cells were then examined for CD19+ B-cell expression and CD14+ cell expression. Representative flow plots shown in (**g**) and (**i**) for B-cell expression and myeloid cell expression, respectively, in each tissue and graphically represented in (**h**) and (**j**). *n* = 3; *p < 0.05

Though the overall reconstitution of human CD45+ cells was largely similar between engraftment in adult and newborn NRG mice, we compared the level of reconstitution of human lymphocytes and myeloid cells between these two methods (Fig. 2b, c, d, and j). There was no significant difference in the levels of human CD14+ myeloid cell reconstitution between engraftment as adults or pups. In the blood, however, humanized mice engrafted as adults had a significantly increased CD19+ B-cell population and a significantly decreased CD3+ T-cell population compared to mice engrafted as pups. When examining the proportion of CD4+ compared to CD8+ T-cells, both methods of human HSC engraftment resulted in a significantly higher proportion of CD4+ T-cells compared to CD8+ T-cells in the spleen, bone marrow, blood, and thymus (Fig. 2f).

### Profile of human lymphocyte and myeloid cell reconstitution is different between immunological tissues

We next examined human lymphocyte and myeloid cell distribution in the spleen, bone marrow, blood, and thymus of both humanized mice. The human CD3+ T-cell population constituted nearly 25 % or more of the overall human CD45+ cell population in the spleen, blood, and thymus (Fig. 2a and b). However, we observed a significantly decreased proportion of human CD3+ T-cells in the bone marrow compared to all other tissues analyzed (3 ± 0.6 %, average ± SD; Fig. 2a and b). In contrast, the CD19+ B-cell population constituted a significant proportion of the human CD45+ cells in both the spleen and bone marrow of mice engrafted as adults and pups, but only in the blood of mice engrafted as adults (Fig. 2i and j). As expected, the thymus contained a significantly decreased level of CD19+ B-cells in comparison to all other tissues analyzed (4.6 ± 3.7 %; Fig. 2g and h). Upon examination of human NK (CD56 + CD3-) and NKT-cells (CD56 + CD3+), we observed a comparable level of NK cells in all tissues between 1 and 4 %, but very low levels of NKT cells in the spleen (0.6 ± 0.2 %), bone marrow (0.4 ± 0.1 %), and blood (0.1 ± 0.01 %; Fig. 2a, c, and d). Interestingly, the largest proportion of NKT cells was observed in the thymus of mice engrafted as either adults or pups (3.3 ± 2.17 %; Fig. 2a and d). In regards to human CD14+ cell reconstitution, the highest levels were found in the bone marrow (8.2 ± 1.1 %), compared to the spleen (0.9 ± 0.2), and thymus (0.2 ± 0.2), but not blood (3.6 ± 0.7; Fig. 2i and j).

### Humanized mice develop mesenteric lymph nodes comprised of human lymphocytes

**Fig. 3:**
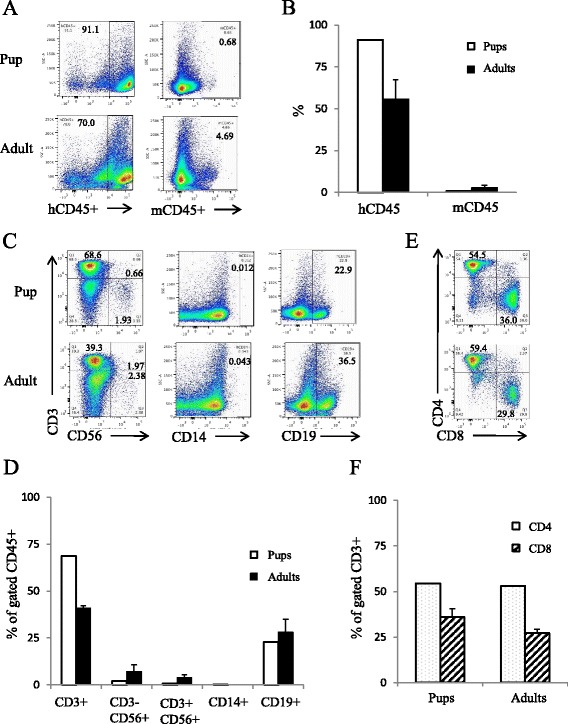
T- and B-cells constitute lymph nodes from human immune cell engrafted pups and adults. Lymph nodes were isolated from adult- and pup-engrafted humanized mice, processed, and examined for mouse CD45 and human CD45, CD3, CD4, CD8, CD56, CD19, and CD14 expression. Events were first analyzed for mouse and human CD45 expression. Representative flow plots displayed in (**a**) and graphically represented in (**b**). Based on human CD45 expression, lymph nodes were then analyzed for CD3, CD56, CD14, and CD19 expression. Representative flow plots shown in (**c**) and stratified by tissue in (**d**). Human CD3 cells were further examined for human CD4 and CD8 expression, shown in (**e**) and (**f**). *n* = 2

There is limited information available on human immune cells in the lymph nodes of humanized mice. Interestingly, the only lymph nodes found in the reconstituted mice were mesenteric lymph nodes. When comparing human and mouse CD45+ levels in the mesenteric lymph nodes of both adult and pup engrafted humanized mice, we observed a large proportion of human CD45+ cells compared to few if any mouse CD45+ cells, as expected (Fig. 3a and b). The lymph nodes were dominated by a CD3+ T-cell compartment, followed by CD19+ B-cell population (Fig. 3c and d). There were small proportions of NK and NKT cells present as well (Fig. 3c and d). Upon further examination of the T-cell compartment, we observed a higher proportion of CD4+ T-cells compared to CD8+ T-cells, similar to observations in other tissues examined (Fig. 3e and f).

### T- and B-cells dominate the significant human immune cell reconstitution in the liver of humanized mice

**Fig. 4:**
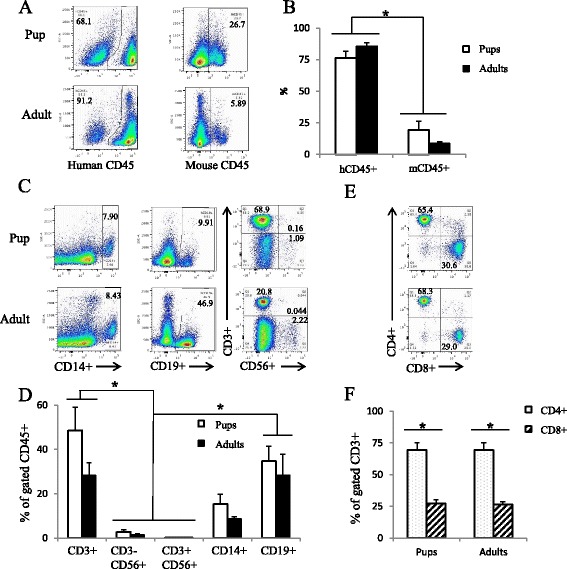
Human immune cell reconstitution in the liver of adult and pup-engrafted humanized mice comprised of T- and B-cells. Livers were isolated from humanized mice 9–28 weeks post transplantation and analyzed for mouse CD45 and human CD45, CD3, CD4, CD8, CD56, CD19, CD14 expression. Events were first analyzed for mouse and human CD45 expression. Representative flow plots displayed in (**a**) and graphically represented in (**b**). Based on human CD45 expression, livers were then analyzed for CD3, CD56, CD14, and CD19 expression. Representative flow plots shown in (**c**) and stratified by tissue in (**d**). Human CD3 cells were further examined for human CD4 and CD8 expression, shown in (**e**) and (**f**). *n* = 3; *p < 0.05

Little is known about human immune cell reconstitution in the livers of humanized mice. At 9 to 28 weeks post intravenous or intrahepatic injection, we observed significantly higher levels of human CD45+ cells (>75 %) compared to mouse CD45+ cells (<20 %) in the livers of both adult and pup engrafted mice (Fig. 4a and b). Upon further examination of the immune subset composition of the human CD45+ population, we found that there were significant CD3+ T-cell and CD19+ B-cells populations residing in the livers of these humanized mice (Fig. 4c and d). Moreover, similar to other tissues examined, we observed a higher proportion of CD4+ T-cells compared to CD8+ T-cells (Fig. 4e). While T- and B-cells dominate the immune cell compartment of the liver, there was a small proportion of CD14+ myeloid cells and an even smaller level of CD56+ NK cells (Fig. 4c and d).

### Human NK cells display a reduced expression of NKp30 and CD11b within the liver of humanized mice engrafted as either adults or newborns

**Fig. 5:**
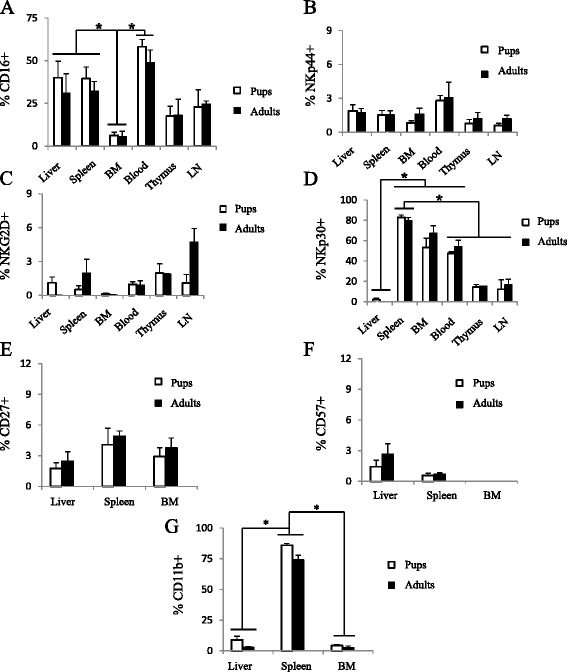
Differential expression of human CD16, NKp30, and CD11b on NK cells in the liver, spleen, BM, blood thymus and liver of CD34+ hematopoietic stem cell-engrafted pups and adult humanized mice. Liver, spleen, bone marrow, blood, thymus, and lymph node were isolated from adult- or pup-engrafted humanized mice 22–28 weeks post-engraftment, processed, and examined for a human CD45, CD3, CD56, CD16, NKp44, NKG2D, NKp30, CD27, CD11b, and CD57. Cells were first gated on human CD45 + cells and subsequently analyzed for human CD3 and CD56 expression. NK cells (CD3-CD56+) from all isolated tissues were then analyzed for CD16, NKp44, NKG2D, and NKp30 expression in (**a**), (**b**), (**c**), and (**d**), respectively. NK cells from liver, spleen, and bone marrow were analyzed for CD27, CD11b, and CD57 expression in (**e**), (**f**), and (**g**), respectively. *n* = 5–8; *p < 0.05

Little is known about NK cell phenotype particularly in liver of humanized mice. There was a significantly decreased expression of CD16 on human NK cells in the bone marrow (6.0 ± 2.4 %) in comparison to the liver (35.4 ± 10.5), spleen (35.9 ± 6.2), and blood (53.6 ± 5.8), but not from thymus (17.8 ± 7.6) and lymph node (23.8 ± 5.9; Fig. 5a). However, when we examined NKp30 expression, there was a significant absence of NKp30 expression on liver NK cells (2.6 ± 1.6) in comparison to the spleen (81.4 ± 2.6), blood (50.8 ± 3.9), and bone marrow (60 ± 8.3) and a significantly increased expression of NKp30 on NK cells in the spleen (Fig. 5d). There was very low, but comparable expression NKp44, NKp46, NKG2D and CD27 on the human NK cells between all tissues examined, including the liver (Fig. 5b, c, and e; NKp46 data not shown). We observed very little expression of CD57 on the human NK cells in all three tissues examined (Fig. 5f). Interestingly, however, there was a significantly higher expression of CD11b on human NK cells in the spleen (80.1 ± 2.5) compared to the liver (5.9 ± 1.7) and bone marrow (3.5 ± 1.0; Fig. 5g).

## Discussion

In this study, we compared human immune cell reconstitution as well as the distribution of various immune subsets between two different methods of HSC engraftment: intravenously in adult NRG mice or intrahepatically in newborn NRG mice. Similar to the findings of Brehm et al. [4], mice engrafted as newborns had higher levels of human CD3+ T-cells and lower levels of CD19+ B-cells in the blood compared to mice engrafted as adults. We observed very little difference in the level of human CD45+ cells in the blood, spleen, bone marrow, thymus, lymph node, or liver between mice engrafted as adults or pups, suggesting that there is very little difference in human immune cell reconstitution between either methods. Furthermore, we observed a long-term reconstitution (up to 28 weeks) of human immune cells in mice engrafted as either adults or newborns. Human T- and B-cell populations were found in all tissues examined, with lower levels of myeloid, NK, and NKT cells, similar to the results of others. Lastly, similar to the human thymus, the humanized mouse thymus contained a high population of human T-cells.

As reported by others, we also observed the development of mesenteric lymph nodes in the humanized mice, engrafted either as adults or pups [5, 6]. Interestingly, we found that the mesenteric lymph nodes were the only lymph nodes to develop in the humanized mice. The presence of microbiota in the gastrointestinal tract likely facilitates the preferential development of mesenteric lymph nodes. It has been previously shown, however, that humanized mice are capable of developing lymph nodes during infection. Kwant-Mitchell et al. [10] found that humanized mice in a Rag2^-/-^γc^-/-^ background that were immunized or infected with herpes simplex virus type 2 developed iliac lymph nodes, whereas naïve mice were only able to develop mesenteric lymph nodes.

Recently, a number of research groups have developed a humanized mouse model in which human hepatocytes have been engrafted into mice, thus providing the opportunity to study human liver tropic pathogens, such as hepatitis B and C in a biological system [7]. Furthermore, double-chimeric humanized mice have been developed, in which both the human immune system and human liver hepatocytes are engrafted onto a mouse background, thereby enabling the study of the human immune response against these pathogens [8, 9]. Human livers contain a distinct T-cell population in the liver, with a higher proportion of CD8+ T-cells over CD4+ T-cells. In our models, we observed a higher ratio of CD4/CD8+ T-cell population. Similar to all other tissues, there were very low levels of human NK and myeloid cells, which has been observed in other studies [7, 11]. Using newborn engraftment, Washburn et al. [7] observed a predominant human T-cell population in the livers of humanized mice as well as small populations of NK cells and plasmacytoid dendritic cells. Likewise, Billerbeck et al. [11] observed high levels of human B- and T-cells in the liver of humanized transgenic A2 mice, in which mice have been genetically modified to express HLA-A2. Indeed, the humanized mice we generated have decreased levels of NK cells, not only in the liver, but in all other tissues we examined.

The immune population within the human liver is largely composed of innate immune cells. Approximately 30–50 % of the immune cells in the human liver are NK cells, which have been observed to express a decreased level of CD16 compared to peripheral blood NK cells [12–14]. It is evident that the current humanized mouse models, in which HSCs are transplanted into immunocompromised murine recipients, are unable to support a identifiable level of human NK cells or other human innate immune cells, likely due to the lack of compatibility between mouse and human cytokines that are required to stimulate innate immune cell development [15]. While researchers have attempted to circumvent the problem by administering specific human cytokines allowing for innate immune cell development, this resulted in abnormally high levels of human cytokines that could cause artificial responses [16–19]. Recently, Rongvaux et al. [20] developed a transgenic mouse model recipient in which they knocked-in five human cytokines into the mouse genome, and found that the human cytokines allowed for the greater development of human monocytes and NK cells in their humanized mouse model. In particular, the generation of human monocytes were able to trans-present human IL-15 to foster the development of NK cells [20]. In double-chimeric mice, the presence of human hepatocytes encouraged the establishment of human Kupffer cells in the liver, which are known to trans-present IL-15 and could stimulate the development of human NK cells [9, 21]. Thus, human hepatocytes may promote a greater level of human NK cells within the liver of double-chimeric mice.

Previous studies have found that human NK cells in these mouse models are not only decreased in number but are also dysfunctional [22]. We found that human NK cells within the bone marrow and liver had a reduced expression of CD16, NKp30 and CD11b, suggesting that these NK cells are less activated and less mature. They also displayed a comparable level of CD16 expression to peripheral blood and splenic NK cells. In contrast, studies examining human liver NK cells found that they express decreased CD16 compared to peripheral blood NK cells [12–14]. Due to the low level of NK cells, however, it would be beneficial to examine NK cell phenotype, maturation, and functionality in a model with a higher frequency of NK cells.

## Conclusions

In conclusion, our study reveals that there is little difference in reconstitution of human immune cells between mice engrafted as adults or newborns. As well, the comprehensive analysis of human immune cell distribution found that each immunological organ we examined had a distinct profile of immune cells. More importantly, human immune cells, particularly NK cells, repopulate the livers and mesenteric lymph nodes of NRG mice and this can provide a relevant pre-clinical model to study the human immune system in the gastrointestinal tract.

## Abbreviations

HSCs, hematopoietic stem cells; NK, natural killer; NRG, NOD-Rag1^-/-^IL2rγ^-/-^; NRG: NOD, non-obese diabetic; PBMCs, peripheral blood mononuclear cells; PBS, phosphate buffered saline; Rag, recombination-activating gene; SCID, severe combined immunodeficiency

## Additional file

Additional file 1: Table S1. Flow cytometry antibody panels. (DOCX 17 kb)
