# Supplementary material for: Reconstitution of immune cell in liver and lymph node of adult- and newborn-engrafted humanized mice
Source: BMC Immunol. 2016 Jun 16;17:18. doi: 10.1186/s12865-016-0157-9 (PMC4910253; doi:10.1186/s12865-016-0157-9)
Supplement: Additional file 1: Table S1. — Flow cytometry antibody panels. (DOCX 17 kb) [file 12865_2016_157_MOESM1_ESM.docx]

Supplementary M&M

Table I: Flow cytometry antibody panels.

| **Panel 1:**  Human immune cell profile | **Antibody** | **Source** | **Clone** |
| --- | --- | --- | --- |
|  | Anti-human CD45 | Biolegend | 2D1 |
|  | Anti-human CD56 | Biolegend | HCD56 |
|  | Anti-human CD4 | Biolegend | A161A1 |
|  | Anti-human CD8 | Biolegend | HITa8 |
|  | Anti-human CD19 | BD Biosciences | HIB19 |
|  | Anti-human CD14 | BD Biosciences | M5E2 |
|  | Anti-mouse CD45 | BD Biosciences | 30-F11 |

| Panel 2:  Activation panel | **Antibody** | **Source** | **Clone** |
| --- | --- | --- | --- |
|  | Anti-human CD45 | Biolegend | 2D1 |
|  | Anti-human CD56 | Biolegend | HCD56 |
|  | Anti-human CD3 | Biolegend | HIT3a |
|  | Anti-human CD16 | BD Biosciences | 3G8 |
|  | Anti-human NKp44 | Biolegend | P44-8 |
|  | Anti-human NKp46 | Biolegend | 9E2 |
|  | Anti-human NKG2D | Biolegend | 1D11 |
|  | Anti-human NKp30 | Biolegend | P30-15 |

| Panel 3:  Maturation panel | **Antibody** | **Source** | **Clone** |
| --- | --- | --- | --- |
|  | Anti-human CD45 | Biolegend | 2D1 |
|  | Anti-human CD56 | Biolegend | HCD56 |
|  | Anti-human CD3 | Biolegend | HIT3a |
|  | Anti-human CD16 | BD Biosciences | 3G8 |
|  | Anti-human CD57 | Biolegend | HCD57 |
|  | Anti-human CD27 | Biolegend | M-T271 |
|  | Anti-human CD11b | Biolegend | CBRM1/5 |
